# Association of childhood-to-adulthood body size change with cancer risk: UK Biobank prospective cohort

**DOI:** 10.1186/s12916-025-04052-8

**Published:** 2025-05-07

**Authors:** Marko Mandic, Fatemeh Safizadeh, Ben Schöttker, Michael Hoffmeister, Hermann Brenner

**Affiliations:** 1 Division of Clinical Epidemiology and Aging Research, German Cancer Research Center (DKFZ), Im Neuenheimer Feld 581, 69120 Heidelberg, Germany; 2 Medical Faculty Heidelberg, Heidelberg University, Heidelberg, Germany; 3 German Cancer Consortium (DKTK), German Cancer Research Center (DKFZ), Heidelberg, Germany

**Keywords:** Obesity; Overweight; Body mass index; Cancer

## Abstract

**Background:**

While excess weight in adulthood and childhood has been associated with increased cancer risk, the link between body size change from childhood to adulthood and cancer risk requires further investigation. We aimed to examine the associations of childhood-to-adulthood body size change with the risk of obesity-related cancers.

**Methods:**

We used data from the UK Biobank, a prospective population-based cohort study. The main exposure was childhood-to-adulthood body size change, constructed from self-reported body size at age 10 (categories: thinner, average, and plumper than average) and measured body mass index (BMI) at recruitment (normal weight, overweight, and obesity). Primary outcome was obesity-related cancer (13 different cancer types).

**Results:**

Among 448,936 participants (mean [SD] age, 56.2 [8.1] years; 240,023 were female [53.5%]) and during a median follow-up of 11.7 years (interquartile range [10.9–12.4]), 21,289 incident obesity-related cancer cases were recorded. Most participants were either overweight (42.6%) or had obesity (24.4%) at recruitment, while only a minority (16.0%) reported to have been plumper than average at age 10. Having a larger body size in childhood was strongly associated with having overweight or obesity in adulthood. Compared to participants with average childhood and normal adulthood body size, participants with overweight or obesity in adulthood had a significantly increased risk of obesity-related cancers, regardless of the childhood body size (adjusted hazard ratios ranged from 1.15 [95% CI, 1.06–1.24] to 1.61 [95% CI, 1.50–1.73]). The strength of the association was mostly determined by adulthood BMI, and similar patterns were observed for colorectal, endometrial, kidney, pancreatic, and esophageal cancer. However, a larger body size in childhood was associated with a lower risk of postmenopausal breast cancer (adjusted hazard ratio, 0.86 [95% CI, 0.79–0.93]).

**Conclusions:**

While larger body size in childhood predisposes individuals to overweight and obesity in adulthood, maintaining a healthy weight in adulthood may help mitigate the risk of obesity-related cancers. Our findings highlight the importance of preventing and reducing overweight and obesity in adulthood for primary cancer prevention.

**Supplementary Information:**

The online version contains supplementary material available at 10.1186/s12916-025-04052-8.

## Background

Adulthood overweight and obesity, commonly defined [1] by a body mass index (BMI) from 25 to 29.9 kg/m^2^ and 30 kg/m^2^ or above, respectively, are established risk factors for a variety of cancers [2]. The prevalence of overweight and obesity has increased rapidly over the last few decades. According to the NCD Risk Factor Collaboration 2024 estimates, there are now around 880 million adults and 159 million children and adolescents living with obesity [3]. In 2016, the International Agency for Research on Cancer (IARC) Working Group concluded that there is sufficient or strong evidence that adulthood excess body weight is a risk factor for esophageal (adenocarcinoma), gastric cardia, colorectal, liver, gallbladder, pancreatic, breast (postmenopausal), endometrial, ovarian, kidney (renal-cell) cancers, and multiple myeloma and meningioma [4]. Recent meta-analyses have further linked excess weight during childhood and adolescence to elevated risk of several cancer types in adulthood [5, 6]. One of the suggested underlying mechanisms of obesity-related carcinogenesis is the continuous release of growth factors, hormones, and pro-inflammatory substances by the fatty tissue [7]. It is therefore plausible to assume that lifetime exposure and body size transitions over time also play a critical role in the risk of obesity-related cancers [8–13]. However, existing studies have primarily focused on cumulative exposure during adulthood, leaving a gap in understanding the link between the change of excess body weight from childhood to adulthood and cancer risk.

Using data from the UK Biobank cohort, we aimed to examine the relationship between childhood and adulthood excess weight and how it relates to the risk of obesity-related cancer in adulthood. To achieve this, we evaluated the association of childhood-to-adulthood body size change with cancer risk.

## Methods

### Study population

The UK Biobank is a prospective population-based cohort, comprising half a million participants aged 40–69 years at baseline, recruited between 2006 and 2010. Details of the rationale, design, and survey methods for the UK Biobank have been described elsewhere [14]. Participants completed a touchscreen questionnaire during the baseline assessment center visit that included questions on socio-demographics, lifestyle, health and medical history, and sex-specific factors. Additionally, several physical measurements including height and weight were performed on the whole cohort during the baseline assessment center visit. All participants provided electronically signed informed consent. Participants were excluded from the analyses if they had a previous cancer diagnosis (except non-melanoma skin cancer) or had missing information on height, weight, or comparative body size at age 10.

### Exposure ascertainment

During the initial assessment visit, trained staff measured standing height using a Seca 202 device. Weight measurements were taken using the Tanita BC-418 MA body composition analyzer [15]. Weight (in kg) was divided by the square of height (in meters) to calculate body mass index (BMI). Three categories (according to the WHO definition) [1] of BMI (normal weight, below 25; overweight, 25 to below 30; and obesity, 30 or higher) were used. Underweight (BMI below 18.5) was not considered a separate category due to the small sample size (0.5% of the study population).

Childhood body size was assessed by asking the participants if they would describe themselves as being “thinner,” “average,” or “plumper” compared to the average 10-year-old child when they were 10 years old.

A new variable “childhood-to-adulthood body size change” was constructed according to participants’ childhood body size and baseline BMI category, resulting in 9 possible categories: average-to-normal weight, average-to-overweight, average-to-obesity, thinner-to-normal weight, thinner-to-overweight, thinner-to-obesity, plumper-to-normal weight, plumper-to-overweight, and plumper-to-obesity.

### Cancer ascertainment and follow-up

Information on cancer incidence (10th revision of the International Statistical Classification of Diseases, ICD-10) is provided by the UK Biobank through linkage to national cancer registries. Thirteen cancer types, identified by IARC (International Agency for Research on Cancer) [4] as obesity-related cancers, were included in the analysis. These included esophageal (adenocarcinoma), gastric cardia, colorectal (CRC), liver, gallbladder, pancreatic, breast (postmenopausal), endometrial, ovarian, kidney (renal-cell), and thyroid cancers, and multiple myeloma and meningioma. This analysis includes complete cancer follow-up data until the 29th of February 2020 for England, the 31st of December 2016 for Wales, and the 31st of January 2021 for Scotland. Given that the censoring dates for England and Scotland were only 1 year apart and that Wales represented just 4% of the UK Biobank participants, these regional differences are unlikely to have influenced the overall analysis. The numbers of cases for the considered cancers are listed, in the order of their frequencies, in Additional file 1: Table S1.

### Statistical analysis

Baseline characteristics of the participants were presented using descriptive statistics. The distribution of adulthood BMI was compared between childhood body size categories. The relationship between childhood body size and the odds of having an adulthood BMI above different overweight/obesity thresholds (i.e., BMI > 25, BMI > 30, BMI > 35, and BMI > 40) was further quantified by odds ratio, using sex- and age-adjusted logistic regression.

The associations of childhood body size, adulthood BMI, and childhood-to-adulthood body size change with obesity-related cancer risk were evaluated using multivariable Cox proportional hazards models. Person-years were calculated from the initial assessment visit until (1) cancer diagnosis; (2) loss to follow-up; (3) death; or (4) end of follow-up, whichever came first. Two adjustment levels were applied. The first model was adjusted for age at baseline (years, continuous) and sex (male, female). The second model (main results) was additionally adjusted for potential confounders identified in the literature: level of education (none, lower academic/professional, higher academic/professional) [16], ethnic background (White, Asian, Black, Mixed, and other) [17], socioeconomic status (Townsend deprivation index, continuous) [18], smoking status (never, former, current) [19], alcohol consumption (never, special occasions only, 1–3 times a month, 1–2 times per week, 3–4 times per week, almost daily or daily) [20], physical activity (International Physical Activity Questionnaire (IPAQ) [21]—low, moderate, high) [20], first-degree family history of breast (no, yes) [22] and colorectal cancer (no, yes) [23], history of bowel cancer screening (no, yes; for obesity-related cancers and CRC only) [24], history of mammography (no, yes; women only; for obesity-related cancers and breast cancer only) [22, 25], hormone replacement therapy (no, yes; women only) [26, 27], fruit intake (pieces per day, continuous) [20], vegetable intake (tablespoons per day, continuous) [20], and red and processed meat intake (never, less than once a week, once a week, ≥ 2 a week) [20]. Given that the changes from the minimally adjusted to fully adjusted models were rather modest, we have not presented the results from the minimally adjusted models. Schoenfeld residuals plots were examined to assess deviations from the proportionality assumption and none was found. Additionally, to avoid the possibility of bias due to prediagnostic weight loss present already at baseline [28], the first 4 years of follow-up were excluded for the second model. This means that the follow-up start was delayed to 4 years after the recruitment, and the participants with the follow-up duration of 4 years or less were removed from the analysis.

Hazard ratios (HRs) and corresponding 95% confidence intervals (CIs) were calculated to estimate the risk of obesity-related cancer per each category for childhood body size, BMI at baseline, and body size change. The reference categories were average, normal weight, and average-to-normal for childhood body size, adulthood BMI, and body size change, respectively. The statistical significance of any interaction between childhood body size and adulthood BMI was assessed via a multivariate Wald test by comparing a model with product terms between the two variables to a model without these terms. The analysis was repeated for obesity-related cancers excluding postmenopausal breast cancer (due to differential patterns of association) and for individual cancer types that had a case count of at least 1000 (before the exclusion of the first 4 years of follow-up), i.e., postmenopausal breast, colorectal, endometrial, kidney, pancreatic, and esophageal cancer.

For covariates with missing values, we performed multiple imputation. All variables included in the second model were included as predictors. We performed 20 iterations and generated 5 imputed data sets (Additional file 1: Table S2). All further analyses were performed with the imputed datasets and the results were combined using Rubin’s rule [29]. Multiple imputation procedures were performed in R using the mice package [30].

We conducted subgroup analyses by stratifying the participants based on sex (male vs female), smoking status (never vs ever smokers), and age at baseline (< 50, ≥ 50 to 59, ≥ 60) to assess potential differences within these subgroups. We evaluated the interactions using the multivariate Wald test by comparing models with and without product terms between pertinent risk factors and childhood body size, adulthood BMI, and body size change.

All statistical analyses were conducted using R version 4.3.2 (R Project for Statistical Computing) [31]. Statistical tests were 2-sided, and an *α* = 0.05.

## Results

**Fig. 1:**
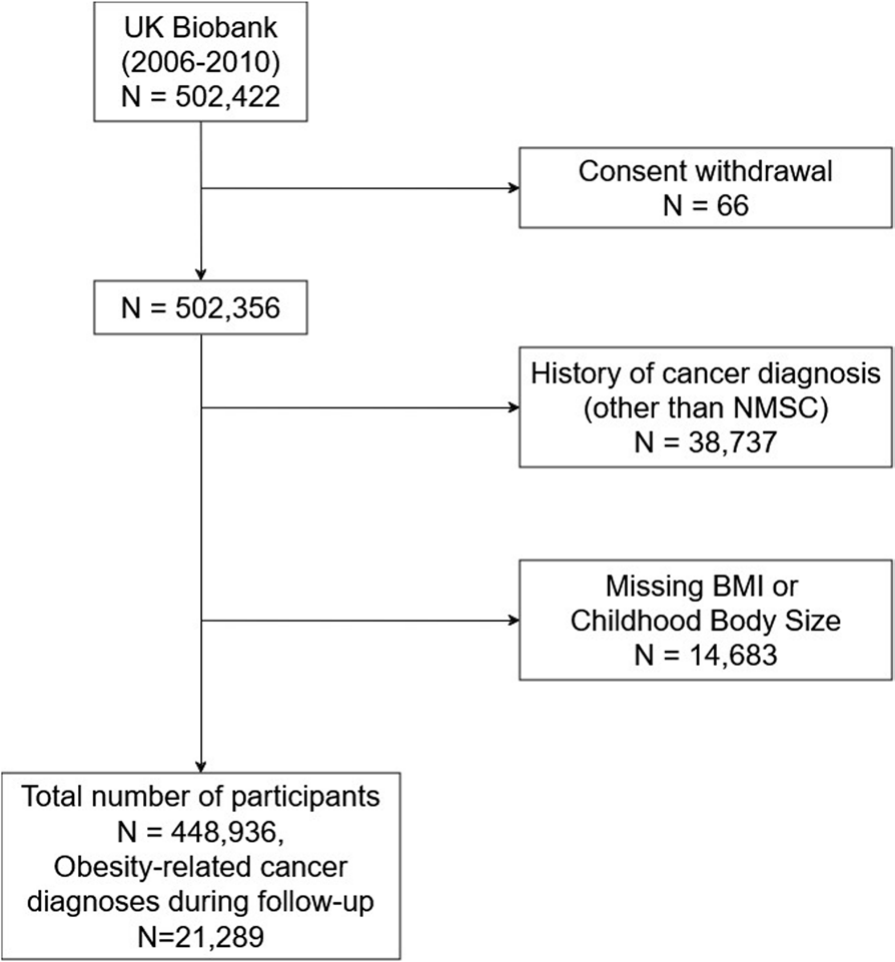
Flow chart showing the selection of the study population

Of the 502,422 participants in the UK Biobank, 66 participants with withdrawn consent, 38,737 with a previous cancer diagnosis, and 14,683 with missing information on childhood body size or adulthood BMI were excluded from the analysis (Fig. 1). The final analyzed dataset included 448,936 participants, of whom 21,289 were diagnosed with obesity-related cancer during a median follow-up time of 11.7 (interquartile range 10.9–12.4) years.

Baseline characteristics of the study population are presented in Table 1. The mean (SD) age at baseline was 56.2 (8.1) years; 240,023 were women (53.5% vs 208,913 men [46.5%]), and 424,234 were White (94.7% vs 24,702 other [5.3%]). At the time of recruitment, 42.6% of participants had overweight and 24.4% had obesity, whereas only a minority (16.0%) reported to have been plumper than average at age 10.

**Table 1:** Baseline characteristics of the study population

**Characteristics**	***N* (col%)**	**Person-years**	***N* cases**	**Incidence/1000 person-years**
**Age at recruitment (years)**				
< 50	109,518 (24.4)	1,285,540	1769	1.4
≥ 50– < 60	151,929 (33.8)	1,740.891	6822	3.9
≥ 60	187,489 (41.8)	2,076,075	12,698	6.1
**Sex**				
Male	208,913 (46.5)	2,372,402	6931	2.9
Female	240,023 (53.5)	2,726,104	14,358	5.3
**Adulthood BMI (kg/m**^**2**^**)**^**a**^				
Normal weight	148,227 (33.0)	1,695,266	6117	3.6
Overweight	191,167 (42.6)	2,173,739	8846	4.1
Obesity	109,542 (24.4)	1,229,501	6326	5.1
**Childhood body size**				
Thinner	148,556 (33.3)	1,696,304	10,661	4.1
Average	227,743 (50.7)	2,590,054	7191	4.2
Plumper	71,637 (16.0)	812,149	3437	4.2
**Body size change**				
Thinner → normal weight	59,987 (13.4)	685,795	2449	3.6
Thinner → overweight	61,767 (13.8)	700,209	3026	4.3
Thinner → obesity	27,802 (6.2)	310,299	1716	5.5
Average → normal weight	74,567 (16.6)	853,253	3098	3.6
Average → overweight	101,467 (22.6)	1,155,456	4599	4.0
Average → obesity	51,709 (11.5)	581,344	2964	5.1
Plumper → normal weight	13,673 (3.0)	156,217	570	3.6
Plumper → overweight	27,933 (6.2)	318,073	1221	3.8
Plumper → obesity	30,031 (6.7)	337,858	1646	4.9
**Ethnicity/race**				
White	423,915 (94.7)	4,818,012	20,540	4.3
Mixed-other	6587 (1.5)	74,056	239	3.2
Asian-Chinese	9988 (2.2)	112,094	302	2.7
Black	6997 (1.6)	78,207	205	2.6
Missing	1449	16,137	64	4.0
**Deprivation index (quartiles)**				
1 (most affluent)	112,097 (25.0)	1,283,045	5376	4.2
2	112,097 (25.0)	1,275,206	5502	4.3
3	112,097 (25.0)	1,269,356	5231	4.1
4 (most deprived)	112,097 (25.0)	1,264,632	5180	4.1
Missing	549	6267	17	2.7
**Qualifications**				
Higher academic/professional	221,333 (49.8)	2,528,962	9761	3.9
Lower academic/vocational	149,332 (33.6)	1,693,881	6805	4.0
None	73,935 (16.6)	827,025	4507	5.4
Missing	4336	48,638	216	4.4
**Smoking status**				
Never	246,570 (55.1)	2,822,337	10,853	3.8
Former	153,757 (34.4)	1,733,483	8160	4.7
Current	47,089 (10.5)	525,767	2214	4.2
Missing	1520	16,916	62	3.7
**Alcohol consumption**				
Never	91,909 (20.5)	1,040,275	4537	4.4
Special occasions only	104,846 (23.4)	1,199,570	4562	3.8
1–3 times a month	116,158 (25.9)	1,324,991	5164	3.9
Once or twice a week	50,001 (11.1)	569,057	2319	4.1
3–4 times a week	50,699 (11.3)	569,774	2822	5.0
Daily or almost daily	34,995 (7.8)	391,148	1865	4.8
Missing	328	3691	20	5.4
**Physical activity (IPAQ groups)**				
Low	63,918 (18.3)	721,525	3114	4.3
Moderate	141,536 (40.6)	1,608,584	6738	4.2
High	142,993 (41.0)	1,627,191	6050	3.7
Missing	100,489	1,141,206	5387	4.7
**Fruit intake (pieces/day)**				
< 2	124,208 (27.7)	1,407,088	5373	3.8
≥ 2– < 5	236,989 (52.9)	2,695,635	11,463	4.3
≥ 5	86,953 (19.4)	987,091	4420	4.5
Missing	786	8691	33	3.8
**Vegetable intake (tablespoons/day)**				
< 3	80,409 (17.9)	915,679	3488	3.8
≥ 3– < 6	226,427 (50.4)	2,575,118	10,924	4.2
≥ 6	139,447 (31.1)	1,578,107	6758	4.3
Missing	2653	29,603	119	4.0
**Red meat intake**				
Never	30,042 (6.8)	342,211	1270	3.7
Less than once a week	151,250 (34.0)	1,718,328	7558	4.4
Once a week	96,580 (21.7)	1,100,942	4553	4.1
≥ 2 times a week	167,118 (37.5)	1,892,858	7712	4.1
Missing	3946	44,168	196	4.4
**Processed meat intake**				
Never	41,367 (9.2)	471,680	1916	4.1
Less than once a week	135,757 (30.3)	1,546,012	6979	4.5
Once a week	130,930 (29.2)	1,486,012	6276	4.2
≥ 2 times a week	140,036 (31.3)	1,487,099	6118	3.9
Missing	846	9404		4.3
**History of CRC screening**				
No	306,980 (69.5)	3,524,205	13,360	3.8
Yes	134,625 (30.5)	1,491,064	7640	5.1
Missing	7331	83,237	289	3.5
**History of mammography (women)**				
No	51,063 (21.3)	596,601	1250	2.1
Yes	188,576 (78.7)	2,125,072	13,095	6.2
Missing	384	4431	13	2.9
**Family history of breast cancer**				
No	402,876 (89.7)	4,578,914	18,527	4.0
Yes	46,060 (10.3)	519,592	2762	5.3
**Family history of CRC**				
No	400,423 (89.2)	4,550,748	18,470	4.1
Yes	48,513 (10.8)	547,756	2819	5.1
**Regular use of NSAIDs/aspirin**				
No	311,225 (69.3)	3,542,998	14,474	4.1
Yes	137,698 (30.7)	1,555,342	6815	4.4
Missing	13	166	0	0
**History of HRT use (women)**				
No	149,064 (62.3)	1,703,622	7575	4.4
Yes	90,146 (37.7)	1,013,364	6738	6.6
Missing	813	9188	45	4.9

Abbreviations: *BMI* body mass index, *CRC* colorectal cancer, *HRT* hormone replacement therapy, *IPAQ* International Physical Activity Questionnaire, *NSAIDs* nonsteroidal anti-inflammatory drugs

^a^BMI (kg/m^2^) was categorized as normal weight (< 25), overweight (≥ 25– < 30), and obesity (≥ 30)

The distribution of adulthood BMI according to childhood body size is shown in Additional file 1: Fig. S1. A major BMI distribution shift towards higher BMIs was observed going from thinner, over average, to plumper childhood body size. Median BMI at recruitment was 26.0, 26.7, and 28.9 kg/m^2^ among participants who reported having been thinner than average, average, and plumper than average at the age of 10, respectively (*p* < 0.001).

**Fig. 2:**
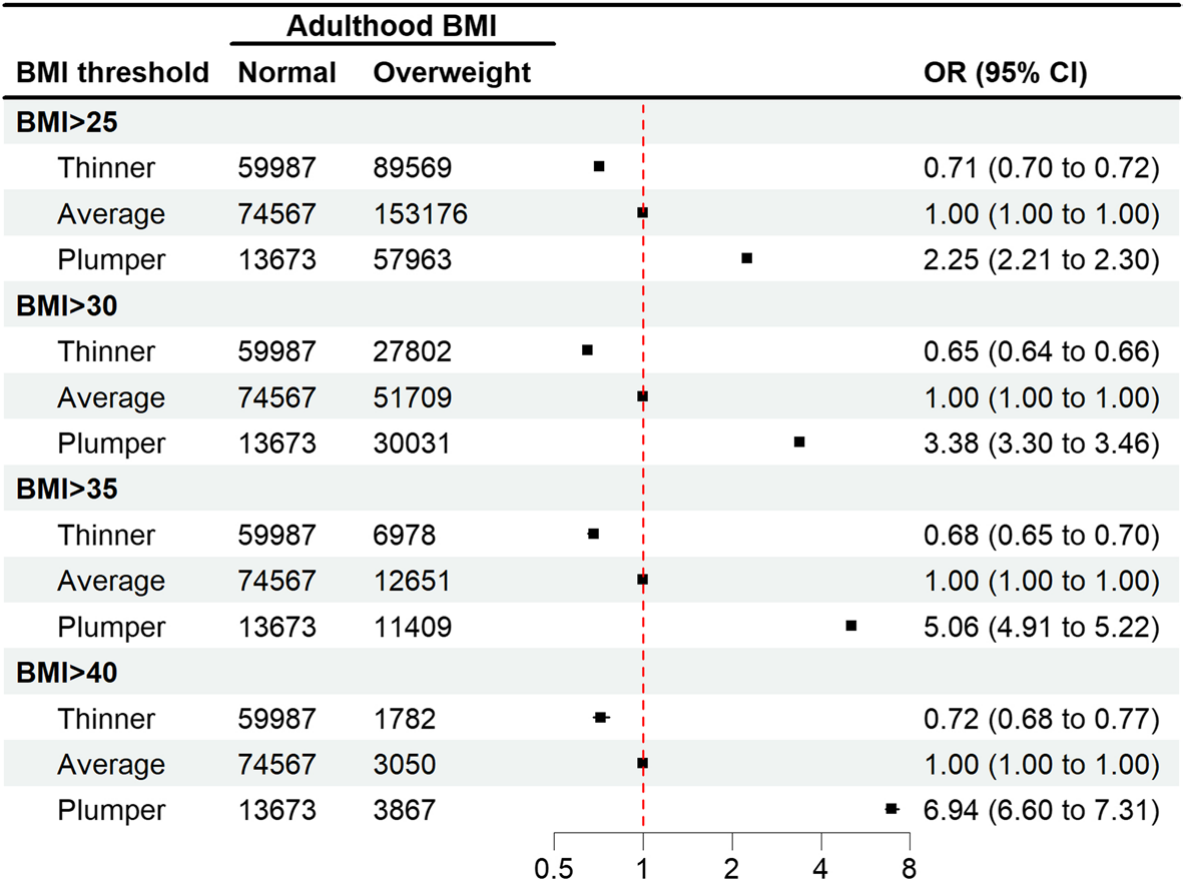
Association of childhood body size with adulthood overweight/obesity

Figure 2 presents the odds ratios for having a BMI above different overweight/obesity thresholds according to the childhood body size. Compared to participants who considered themselves to have had an average body size at age 10, participants who marked “thinner” had significantly lower odds of having high adulthood BMI (ORs ranging from 0.65 to 0.72). In contrast, participants who reported being plumper at age 10 were more likely to have overweight or obesity in adulthood, with ORs rising from 2.25 (95% CI 2.21 to 2.30) for having a BMI > 25 kg/m^2^ to 6.94 (95% CI 6.60 to 7.31) for having a BMI > 40 kg/m^2^.

**Fig. 3:**
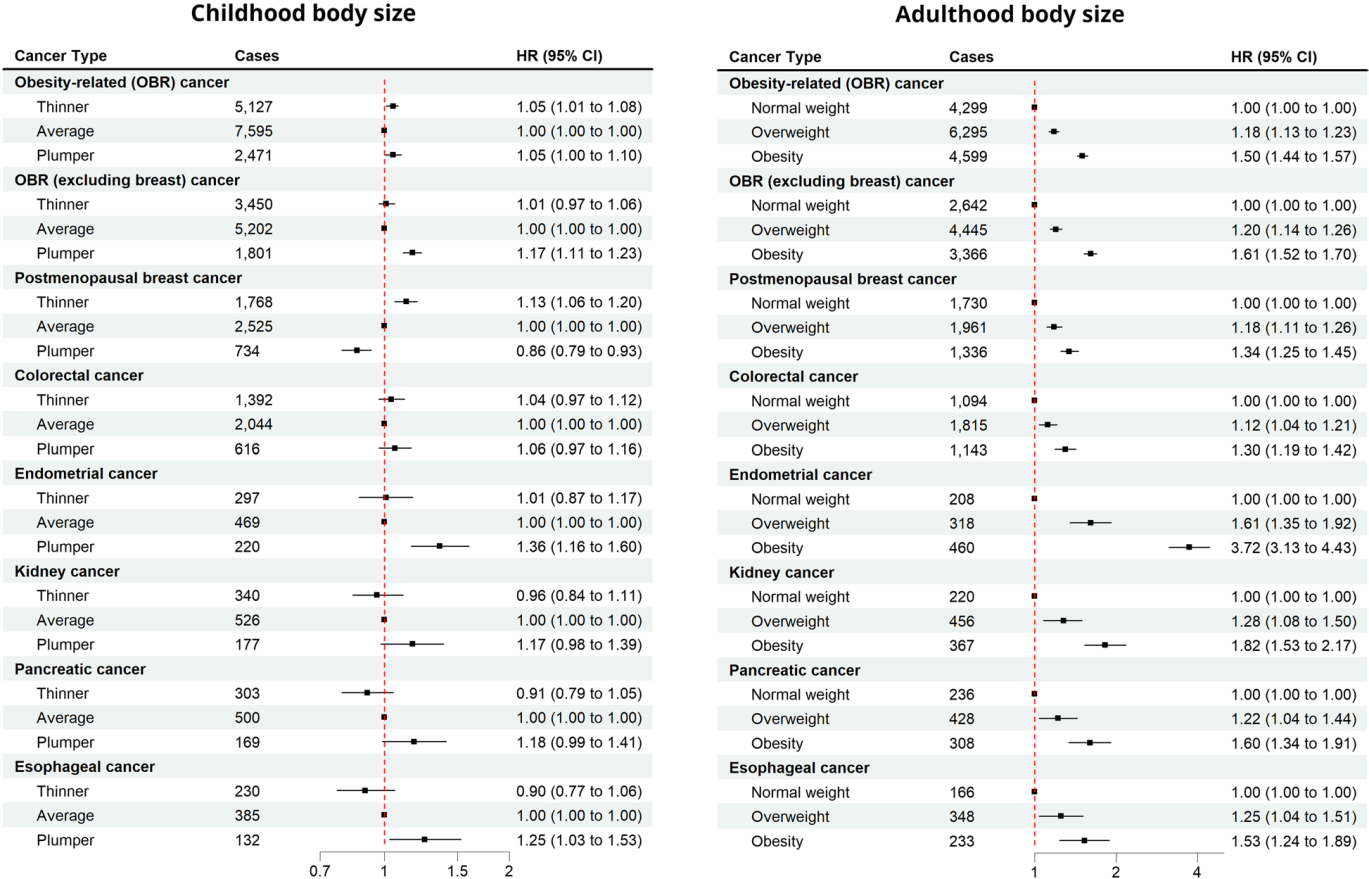
Association of childhood body size and adulthood BMI with obesity-related cancer risk

Figure 3 shows the association of childhood body size and adulthood BMI with obesity-related cancer risk. Compared to self-reported average body size at age 10, self-reported thinner and plumper body size were associated with a modest increase in obesity-related cancer risk (HR 1.05, 95% CI 1.01 to 1.08 and HR 1.05, 95% CI 1.00 to 1.10, respectively). However, after exclusion of postmenopausal breast cancer, no association with thinner body size (HR 1.05, 95% 0.97–1.06), and stronger positive association with plumper body size (HR 1.17, 95% 1.11–1.23) was found for obesity-related cancer risk. Being plumper at age 10 was positively associated with an increased risk of endometrial (HR 1.36, 95% CI 1.16 to 1.60) and esophageal cancer (HR 1.25, 95% CI 1.03 to 1.53) and with a lower postmenopausal breast cancer risk (HR 0.86, 95% CI 0.79 to 0.93). Adulthood overweight and obesity were associated with an 18% and 51% increased risk of obesity-related cancers (HR 1.18, 95% CI 1.13 to 1.23 and HR 1.51, 95% CI 1.44 to 1.57). Strong positive associations were observed for all of the common obesity-related cancer types, with HRs ranging from 1.12 to 1.61 for overweight and from 1.30 to 3.71 for obesity. With a HR of 3.72 (95% CI 3.13–4.43), the by far strongest association was seen between obesity and endometrial cancer. No significant interaction between childhood body size and adulthood BMI was observed for obesity-related cancer, nor for any of the selected cancer types.

Table 2 shows the association of childhood-to-adulthood body size change with the risk of obesity-related cancer and selected cancer types. Compared to the average-to-normal group, all other childhood-to-adulthood body size change patterns (except plumper-to-normal) had an increased risk of obesity-related cancer, with the HRs ranging from 1.07 to 1.61. With the exception of postmenopausal breast cancer, similar patterns were observed for all of the six most common obesity-related cancer types, with the risk mostly being determined by adulthood overweight and obesity. Lower postmenopausal breast cancer risk was observed for patterns starting with being plumper compared to starting with being thinner or average at age 10.

**Table 2:** Body size change and obesity-related cancer risk

	**Thinner → normal weight**	**Thinner → overweight**	**Thinner → obesity**	**Average → normal weight**	**Average → overweight**	**Average → obesity**	**Plumper → normal weight**	**Plumper → overweight**	**Plumper → obesity**
**All obesity-related cancers** (*N* = 15,193)									
No. cases	1746	2141	1240	2160	3276	2159	393	1878	1200
Incidence rate^a^	389.7	469.8	618.4	387,3	434.8	572.8	385.0	423.4	547.8
HR (95% CI)^b^	1.07 (1.00–1.14)	1.28 (1.21–1.36)	1.61 (1.50–1.73)	Reference	1.19 (1.12–1.26)	1.53 (1.44–1.63)	0.99 (0.89–1.10)	1.15 (1.06–1.24)	1.52 (1.41–1.63)
**Obesity-related cancers, excluding breast cancer** (*N* = 10,453)									
No. cases	1076	1479	895	1311	2337	1554	255	629	917
Incidence rate^a^	237.7	321.1	440.5	232.5	307.3	407.4	247.2	300.5	414.5
HR (95% CI)^b^	1.04 (0.96–1.12)	1.24 (1.15–1.34)	1.66 (1.52–1.82)	Reference	1.21 (1.13–1.29)	1.58 (1.46–1.70)	1.12 (0.98–1.28)	1.26 (1.15–1.39)	1.76 (1.61–1.92)
**Post-menopausal breast cancer** (*N* = 5027)									
No. cases	695	703	370	889	988	648	146	270	318
Incidence rate^a^	260.3	352.9	360.4	234.7	288.3	343.1	187.5	230.1	250.7
HR (95% CI)^b^	1.12 (1.02–1.24)	1.43 (1.29–1.58)	1.54 (1.36–1.75)	Reference	1.17 (1.07–1.28)	1.45 (1.31–1.61)	0.82 (0.69–0.98)	0.98 (0.86–1.13)	1.14 (1.00–1.29)
**Colorectal cancer** (*N* = 4052)									
No. cases	462	626	304	536	936	545	96	226	294
Incidence rate^a^	101.5	135.0	148.0	94.5	125.7	141.5	92.5	107.3	131.5
HR (95% CI)^b^	1.07 (0.94–1.21)	1.21 (1.08–1.36)	1.37 (1.18–1.58)	Reference	1.14 (1.02–1.27)	1.31 (1.16–1.48)	1.07 (0.86–1.33)	1.09 (0.93–1.27)	1.38 (1.20–1.60)
**Endometrial cancer** (*N* = 986)									
No. cases	84	103	110	96	169	204	28	46	146
Incidence rate^a^	31.0	50.6	105.1	25.0	48.5	106.2	35.6	38.7	114.0
HR (95% CI)^b^	1.26 (0.94–1.69)	1.95 (1.47–2.58)	4.13 (3.11–5.48)	Reference	1.86 (1.45–2.40)	4.10 (3.19–5.25)	1.49 (0.98–2.27)	1.57 (1.10–2.23)	4.65 (3.57–6.05)
**Kidney cancer** (*N* = 1043)									
No. cases	109	87	24	254	140	62	163	113	91
Incidence rate^a^	19.1	19.0	23.0	33.0	30.0	29.3	42.1	54.8	40.5
HR (95% CI)^b^	0.94 (0.71–1.24)	1.18 (0.91–1.52)	2.11 (1.61–2.77)	Reference	1.33 (1.06–1.67)	1.67 (1.31–2.14)	1.37 (0.88–2.14)	1.34 (0.98–1.84)	1.81 (1.36–2.40)
**Pancreatic cancer** (*N* = 972)									
No. cases	103	132	68	110	239	151	23	57	89
Incidence rate^a^	22.5	28.3	32.9	19.3	31.0	38.9	22.1	26.9	39.6
HR (95% CI)^b^	1.16 (0.89–1.52)	1.23 (0.95–1.59)	1.41 (1.04–1.93)	Reference	1.38 (1.10–1.74)	1.75 (1.36–2.26)	1.25 (0.80–1.97)	1.33 (0.96–1.83)	2.03 (1.53–2.70)
**Esophagus** (*N* = 747)									
No. cases	64	108	58	81	189	115	51	58	60
Incidence rate^a^	14.0	23.1	28.1	14.2	24.5	29.7	20.1	14.1	26.7
HR (95% CI)^b^	0.94 (0.68–1.31)	1.19 (0.89–1.60)	1.45 (1.03–2.05)	Reference	1.29 (0.99–1.68)	1.58 (1.18–2.11)	1.67 (1.03–2.70)	1.50 (1.05–2.13)	1.69 (1.21–2.38)

Abbreviations: *CI* confidence interval, *CRC* colorectal cancer, *HR* hazard ratio, *HRT* hormone replacement therapy, *NSAIDs* nonsteroidal anti-inflammatory drugs

^a^Incidence rate per 100,000 person-years

^b^Model adjusted for age, sex, ethnicity, height, Townsend deprivation index, education, smoking, alcohol consumption, red and processed meat consumption, 1st-degree family history of CRC and breast cancer, previous CRC screening (for obesity-related cancers and CRC), previous mammography (women only; for obesity-related cancers and breast cancer), HRT (women only), fruit and vegetable intake, NSAIDs use, and physical activity

Table 3 displays subgroup analyses based on sex, showing the associations of childhood body size, adulthood BMI, and body size change with obesity-related cancer (occurring in both sexes: colorectal cancer, kidney cancer, pancreatic cancer, esophageal cancer, multiple myeloma, liver cancer, thyroid cancer, stomach cancer, gallbladder cancer, and meningioma) risk. Significant interactions were found between sex and adulthood BMI and body size change. Having a plumper childhood body size was associated with a 14% (5 to 23%) and 18% (8 to 29%) increased risk in men and women, respectively, and no significant associations were found for a thinner childhood body size. Adulthood overweight and obesity were more strongly associated with obesity-related cancer in men than women. Patterns ending with overweight and obesity in adulthood were associated with obesity-related cancer risk, with the associations being stronger in men. Patterns ending with normal weight in adulthood were not associated with cancer risk, except for plumper-to-normal weight in women, where a 21% (95% 1–45%) risk increase was observed. Table S3 (see Additional file 1) shows that the associations were generally somewhat stronger among never-smokers than ever-smokers, and the interaction was significant only for body size change (*p* interaction = 0.022). Table S4 (see Additional file 1) shows the association of childhood body size, adulthood BMI, and body size change according to age groups at baseline. While the patterns were similar for groups 50–59 years and ≥ 60 years, associations of childhood body size, adulthood BMI, and body size change with obesity-related cancer were generally weaker for the participants younger than 50 years at baseline. However, no significant interactions were observed.

**Table 3:** Sex-specific analysis—association of childhood body size, adulthood BMI, and body size change with obesity-related cancer risk*

	**Men**		**Women**		***p* interaction**^**b**^
	***N* cases**	**HR**^**a**^** (95% CI)**	***N* cases**	**HR**^**a**^** (95% CI)**	
**Childhood body size**					
Thinner	1750	0.97 (0.91–1.03)	1184	1.07 (0.99–1.15)	0.15
Average	2649	Reference	1765	Reference	
Plumper	757	1.14 (1.05–1.23)	704	1.18 (1.08–1.29)	
**Adulthood BMI**					
Normal weight	966	Reference	1232	Reference	< 0.001
Overweight	2498	1.26 (1.17–1.35)	1379	1.10 (1.02–1.19)	
Obesity	1692	1.66 (1.53–1.80)	1042	1.31 (1.20–1.43)	
**Body size change**					
Thinner → normal weight	442	0.98 (0.86–1.11)	468	1.10 (0.98–1.24)	< 0.001
Thinner → overweight	855	1.24 (1.11–1.39)	433	1.17 (1.04–1.33)	
Thinner → obesity	453	1.62 (1.42–1.85)	283	1.45 (1.26–1.68)	
Average → normal weight	468	Reference	615	Reference	
Average → overweight	1346	1.22 (1.10–1.37)	711	1.15 (1.03–1.28)	
Average → obesity	835	1.60 (1.43–1.80)	439	1.27 (1.12–1.44)	
Plumper → normal weight	56	0.96 (0.72–1.26)	149	1.21 (1.01–1.45)	
Plumper → overweight	297	1.30 (1.12–1.50)	235	1.19 (1.02–1.38)	
Plumper → obesity	404	1.72 (1.51–1.97)	320	1.51 (1.32–1.74)	

Abbreviations: *BMI* body mass index, *CI* confidence interval, *CRC* colorectal cancer, *HR* hazard ratio, *HRT* hormone replacement therapy, *NSAIDs* nonsteroidal anti-inflammatory drugs

^*^Obesity-related cancers occurring in both sexes: colorectal cancer, kidney cancer, pancreatic cancer, esophageal cancer, multiple myeloma, liver cancer, thyroid cancer, stomach cancer, gallbladder cancer, and meningioma

^a^Model adjusted for age, sex, ethnicity, height, Townsend deprivation index, education, smoking, alcohol consumption, red and processed meat consumption, 1st-degree family history of CRC and breast cancer, previous CRC screening (for obesity-related cancers and CRC), HRT (women only), fruit and vegetable intake, NSAIDs use, and physical activity

^b^Multivariate Wald test comparing the model with and without product terms

## Discussion

In this large population-based cohort study with 448,936 participants and 21,289 obesity-related cancer cases, we evaluated the relationship of self-reported childhood body size with adulthood body size, and how change of body size from childhood to adulthood is associated with the risk of obesity-related cancer. Compared to participants who had an “average” body size at age 10, participants who were “plumper” at age 10 were considerably more likely to have overweight or obesity in adulthood. Interestingly, however, despite differences in body size at age 10, adults who had overweight or obesity faced similar increased obesity-related cancer risks. This was applicable for colorectal, endometrial, kidney, pancreatic, and esophageal cancer, but not for postmenopausal breast cancer where a larger body size at age 10 was associated with a reduced risk. The observed patterns suggest that maintaining a healthy body weight in adulthood could potentially counteract the negative health impacts associated with childhood obesity.

Adulthood overweight and obesity are established risk factors for many cancer types [2, 4]. Recent reviews have corroborated the association between childhood and adolescent obesity and an increased risk of cancer in adulthood, albeit with a body of evidence not as comprehensive or conclusive as that supporting the association between excess weight in adulthood and cancer risk [5, 6]. Biological mechanisms through which excess weight has been linked to carcinogenesis include alterations in sex hormone and adipokine levels, changes in the insulin and insulin-like growth factor signaling, and chronic inflammation [7]. Individuals who have been exposed to excess body weight earlier in life and for longer periods have therefore been more exposed to the aforementioned adverse metabolic and hormonal changes leading to increased cancer risk. Indeed, several recent studies have shown that measures that capture the duration and severity of exposure to excess weight (analogous to pack-years for smoking) are associated with several cancer types [8–10, 12, 13]. Nonetheless, these studies have mostly focused on excess weight exposure from late adolescence or early adulthood onward, not considering childhood overweight and obesity.

In our analysis, we have observed a dose–response relationship between childhood body size and adulthood excess adiposity, with people who were plumper at age 10 being more than two times more likely to have BMI > 25, more than three times to have BMI > 30, five times to have BMI > 35, and almost seven times more likely to have severe obesity (BMI > 40), compared to people with average body size at age 10. An inverse trend was observed for people who were thinner at age 10, who were around 30% less likely to have overweight or obesity in adulthood.

A 2023 systematic review and meta-analysis found significant positive associations between childhood and adolescent obesity with colorectal and pancreatic cancer in both sexes and ovarian cancer in women [6]. Another analysis of childhood BMI trajectories of 301,927 children aged 6–15 years revealed increased rates of adult obesity-related cancers (excluding breast cancer) for “overweight” and “obesity” trajectories (compared to “average” trajectory), with incidence rate ratios of 1.27 (95% CI 1.17–1.38) and 1.79 (95% CI 1.53–2.08), respectively [32]. In our analysis, we found modest positive associations of “thinner” (HR 1.04, 95% 1.01–1.08) and “plumper” (HR 1.05, 95% 1.00–1.10) childhood body sizes with obesity-related cancer risk. However, after exclusion of postmenopausal breast cancer, being “thinner” at age 10 showed no association (HR 1.01, 95% 0.97–1.05), while being “plumper” showed a stronger positive association (HR 1.17, 95% 1.11–1.24) with obesity-related cancer. This effect is likely due to a well-documented [32–35] (but poorly understood) inverse association of excess childhood adiposity and (postmenopausal) breast cancer, which in our study comprises about half of the obesity-related cancer cases in women. For adulthood overweight and obesity, we observed strong associations with obesity-related cancer risk (18% and 51%, respectively), with relative risk estimates similar to those found in previous research [4].

For childhood-to-adulthood body size change, patterns that ended in normal weight in adulthood were associated with the lowest risk. Higher risks (15 to 28%) were observed for patterns ending with adulthood overweight, and the highest risk (52 to 61%) in patterns ending in obesity. This, along with the lack of interaction between childhood body size and adulthood BMI, suggests that it is the adulthood body size that is the main determinant of obesity-related cancer risk. Only a few studies have looked into the relationship between different life-course trajectories (with childhood period being included) of body size or adiposity with cancer risk, most of which focused on breast cancer [33–37]. The most comprehensive evidence comes from a 2016 study by Song and colleagues [37] that investigated the association of 5 identified body shape trajectories (lean-stable, lean-moderate increase, lean-marked increase, medium-stable, and heavy-stable/increase) from age 5 to age 60 with cancer risk. Compared to the lean-stable group, women who had experienced weight gain in later life (lean-moderate increase, lean-marked increase, and heavy-stable/increase) had a significantly higher obesity-related cancer risk (HRs from 1.17 to 1.39), whereas no association for medium-stable women was observed (HR 1.03, 95% CI 0.95 to 1.12). In men, compared to the lean-stable group, an increase in obesity-related cancer risk was observed for all other four trajectories (HR from 1.09 to 1.17), though the associations were not statistically significant. These results are consistent with our findings, with lean-stable being comparable to thinner-to-normal or average-to-normal pattern, and “moderate” and “marked” increase being comparable to having overweight and obesity in adulthood, respectively.

Different patterns of association were observed across selected cancer types and sex and smoking subgroups. As mentioned, we observed an inverse association between childhood body size and postmenopausal breast cancer risk. Compared to women with average body size at age 10, thinner women had a 13% (95% CI 6 to 20%) increased, and plumper women had a 14% (95% CI − 7 to − 21%) decreased risk of postmenopausal breast cancer. Similarly, compared to the average-to-normal body size group, patterns starting with being plumper at age 10 showed inverse or no association, and patterns starting with being thinner at age 10 showed strong positive associations with postmenopausal breast cancer risk. One of the suggested mechanisms is that high adiposity in childhood leads to a reduction in breast tissue density, which in turn reduces the risk of postmenopausal breast cancer [38]. Our results corroborate the findings of two previous studies [33, 37] investigating the association of BMI trajectories across the life course with postmenopausal breast cancer. As suggested previously [33], a woman’s lifetime body size history may be considerably more predictive of postmenopausal breast cancer risk than looking at childhood or adulthood body size alone. This is further evidenced by the results from previous meta-analyses which have shown that weight gain, rather than weight or BMI at a single point in time, better captures postmenopausal breast cancer risk [35, 39, 40]. Of other selected cancer types, we found the strongest association for endometrial cancer, where women with body size patterns ending in obesity had more than four-fold higher risk compared to the average-to-normal group. For pancreatic and esophageal cancer, we have observed that associations for patterns ending with obesity seem to have a dose–response dependency on childhood body size (larger childhood body size—higher risk). However, again, no significant interactions between childhood and adulthood body size were found.

A notable strength of this study is the UK Biobank’s population-based prospective design with a long follow-up, a large number of participants and incident cases, and the availability of information on a wide range of potential confounders. Nonetheless, there are also some limitations. First, the “healthy volunteer” bias has been observed in the UK Biobank [41]. The UK Biobank participants are less likely to have obesity, to smoke, to drink regularly, and have fewer self-reported diseases. Second, while the baseline BMI was assessed through objective measures, it is important to note that the self-reported “comparative body size at age 10” introduces subjectivity and is susceptible to recall bias, which might be nondifferential with respect to adulthood weight and related cancer risk. However, recent systematic reviews have found overall good agreement between self-reported and measured body weight in early life [42, 43]. The substantially higher proportion of participants reporting having been thinner rather than plumper than average at age 10 (33.3% versus 16.0%) may be an indication of selective participation in the UK Biobank, underreporting of childhood weight, or some combination of both. Third, due to the lack of repeated body size measures, we were unable to capture weight fluctuations between the two time points. Fourth, due to the lack of information, we were unable to consider participants’ early life factors that may confound the associations with childhood body size. Finally, the predominantly White composition (95%) of the UK Biobank participants imposes constraints on the applicability of our conclusions to non-White demographic groups.

## Conclusions

In summary, our study examining the association of childhood-to-adulthood body size change with obesity-related cancer risk suggests that, while excess weight in childhood may play a role for certain cancer types, adulthood overweight and obesity are the primary factors influencing the risk of obesity-related cancer. For postmenopausal breast cancer, a larger body size during childhood appears to have a protective influence, contrasting with the patterns observed in other obesity-related cancers. Our results are in concordance with the findings from previous studies, which are notably sparse on this topic. Further research using objective, repeated measures of adiposity across the lifespan and including biomarkers indicative of potential underlying mechanisms are needed to deepen our understanding of the impact of excess weight on cancer risk. Notwithstanding the need for such further research, major efforts are needed to halt and reverse the ongoing global increase in obesity prevalence which otherwise may further accelerate the increasing cancer burden due to demographic aging in many countries.

Supplementary information.

Additional file 1: Table S1 List of obesity-related cancer types (ICD-10) included in this analysis. Table S2 Baseline characteristics of the study population before and after multiple imputation. Fig. S1 Adulthood BMI according to childhood body size. Table S3 Subgroup analysis based on smoking—association of childhood body size, adulthood BMI, and body size change with obesity-related cancer risk. Table S4 Age-subgroup analysis—association of childhood body size, adulthood BMI, and body size change with obesity-related cancer risk.

## Supplementary Information

Supplementary Material 1.

## Data Availability

Data was reused with the permission of the UK Biobank. This work used data provided by patients and collected by the NHS as part of their care and support. The UK Biobank is an open-access resource and bona fide researchers can apply to use the UK Biobank dataset by registering and applying at https://www.ukbiobank.ac.uk/enableyourresearch/apply-for-access. The data and analysis codes used for this study are going to be available on the UK Biobank website for registered researchers at the UK Biobank and an application fee.

## Abbreviations

BMI: Body mass index
CI: Confidence interval
CRC: Colorectal cancer
HR: Hazard ratio
HRT: Hormone replacement therapy
IARC: International Agency for Research on Cancer
IPAQ: International Physical Activity Questionnaire
NSAIDs: Nonsteroidal anti-inflammatory drugs
OR: Odds ratio
SD: Standard deviation
