# Supplementary material for: Association of childhood-to-adulthood body size change with cancer risk: UK Biobank prospective cohort
Source: BMC Med. 2025 May 7;23:268. doi: 10.1186/s12916-025-04052-8 (PMC12060570; doi:10.1186/s12916-025-04052-8)
Supplement: Supplementary file 1 — Supplementary Material 1. [file 12916_2025_4052_MOESM1_ESM.docx]

**Association of childhood-to-adulthood body size change with cancer risk:**

**UK Biobank prospective cohort**

**Table S1**. List of obesity-related cancer types (ICD-10) included in this analysis.

**Table S2**. Baseline characteristics of the study population before and after multiple imputation.

**Figure S1**. Adulthood BMI according to childhood body size.

**Table S3**. Subgroup analysis based on smoking – Association of childhood body size, adulthood BMI, and body size change with obesity-related cancer risk.

**Table S4**. Age-subgroup analysis - Association of childhood body size, adulthood BMI, and body size change with obesity-related cancer risk.

**Table S1.** List of obesity-related cancer types (ICD-10) included in this analysis.

|  |  | **Incident cases [N]** | |
| --- | --- | --- | --- |
| **Cancer type** | **ICD-10 code** | **Full cohort** | **First 4 years of follow-up excluded** |
| Breast (postmenopausal*) | C50.0-C50.6, C50.8, C50.9 | 7,279 | 5,027 |
| Colorectum | C18.0- C18.9, C19, C20 | 5,706 | 4,052 |
| Endometrium | C54.1 | 1,430 | 986 |
| Kidney (renal-cell) | C64 | 1,404 | 1,043 |
| Pancreas | C25.0-C25.4, C25.7, C25.8, C25.9 | 1,244 | 972 |
| Esophagus  (adenocarcinoma) | C15.0-C15.5, C15.8, C15.9 | 1,006 | 747 |
| Ovary | C56 | 953 | 642 |
| Multiple myeloma | C90.0 | 851 | 668 |
| Liver | C22.0, C22.1, C22.3, C22.4, C22.7, C22.9 | 620 | 489 |
| Thyroid | C73 | 428 | 304 |
| Stomach (cardia) | C16.0 | 273 | 193 |
| Gallbladder | C23 | 111 | 78 |
| Meningioma | C70.0, C70.9 | 13 | 9 |
| Total |  | 21,289**^1^** | 15,193^1^ |

*Breast cancers were considered postmenopausal if either of the following conditions was met: reported menopause or history of bilateral oophorectomy at baseline, or age ≥55 years at the time of breast cancer diagnosis.

^1^ The addition of incident cancer cases for each cancer type exceeds the total number of cases due to multiple incident cancers diagnosed on the same date for a number of participants.

**Table S2.** Baseline characteristics of the study population before and after multiple imputation.

| **Characteristic** | **N Missing** | **Before imputation** | **After imputation** | | | | |
| --- | --- | --- | --- | --- | --- | --- | --- |
|  |  |  | **Set 1** | **Set 2** | **Set 3** | **Set 4** | **Set 5** |
| **Ethnicity/Race, N (%)** | 1,449 |  |  |  |  |  |  |
| White |  | 423,915 (94.4) | 425,267 (94.7) | 425,232 (94.7) | 425,255 (94.7) | 425,250 (94.7) | 425,257 (94.7) |
| Mixed-Other |  | 6,587 (1.5) | 6,601 (1.5) | 6,613 (1.5) | 6,609 (1.5) | 6,622 (1.5) | 6,609 (1.5) |
| Asian-Chinese |  | 9,988 (2.2) | 10,039 (2.2) | 10,047 (2.2) | 10,043 (2.2) | 10,031 (2.2) | 10,040 (2.2) |
| Black |  | 6,997 (1.6) | 7,029 (1.6) | 7,044 (1.6) | 7,029 (1.6) | 7,033 (1.6) | 7,030 (1.6) |
| **Qualifications, N (%)** | 4,336 |  |  |  |  |  |  |
| Higher academic/professional |  | 221,333 (49.8) | 223,095 (49.7) | 223,090 (49.7) | 223,093 (49.7) | 223,095 (49.7) | 223,126 (49.7) |
| Lower academic/vocational |  | 149,332 (33.3) | 150,926 (33.6) | 150,979 (33.6) | 150,959 (33.6) | 150,959 (33.6) | 150,906 (33.6) |
| None |  | 73,935 (16.6) | 74,915 (16.7) | 74,867 (16.7) | 74,884 (16.7) | 74,882 (16.7) | 74,904 (16.7) |
| **Deprivation index (cont.), mean (SD)** | 549 | -1.3 (3.1) | -1.3 (3.1) | -1.3 (3.1) | -1.3 (3.1) | -1.3 (3.1) | -1.3 (3.1) |
| **Smoking status, N (%)** | 1,520 |  |  |  |  |  |  |
| Never |  | 246,570 (55.1) | 247,336 (55.1) | 247,336 (55.1) | 247,356 (55.1) | 247,308 (55.1) | 247,354 (55.1) |
| Former |  | 153,757 (34.4) | 154,316 (34.4) | 154,316 (34.4) | 154,317 (34.4) | 154,357 (34.4) | 154,305 (34.4) |
| Current |  | 47,089 (10.5) | 47,284 (10.5) | 47,284 (10.5) | 47,263 (10.5) | 47,271 (10.5) | 47,277 (10.5) |
| **Alcohol consumption, N (%)** | 328 |  |  |  |  |  |  |
| Never |  | 91,909 (20.5) | 91,956 (20.5) | 91,965 (20.5) | 91,956 (20.5) | 91,961 (20.5) | 91,956 (20.5) |
| Special occasions only |  | 104,846 (23.4) | 104,904 (23.4) | 104,911 (23.4) | 104,912 (23.4) | 104,912 (23.4) | 104,910 (23.4) |
| 1-3 times a month |  | 116,158 (25.9) | 116,234 (25.9) | 116,234 (25.9) | 116,241 (25.9) | 116,237 (25.9) | 116,244 (25.9) |
| Once or twice a week |  | 50,001 (11.1) | 50,039 (11.1) | 50,038 (11.1) | 50,043 (11.1) | 50,033 (11.1) | 50,042 (11.1) |
| 3-4 times a week |  | 50,699(11.3) | 50,749 (11.3) | 50,744 (11.3) | 50,751 (11.3) | 50,737 (11.3) | 50,750 (11.3) |
| Daily or almost daily |  | 34,995 (7.8) | 35,054 (7.8) | 35,044 (7.8) | 35,033 (7.8) | 35,056 (7.8) | 35,034 (7.8) |
| **Physical activity (IPAQ groups), N (%)** | 100,489 |  |  |  |  |  |  |
| Low |  | 63,918 (18.3) | 82,957 (18.5) | 83,102 (18.5) | 82,948 (18.5) | 83,148 (18.5) | 82,931 (18.5) |
| Moderate |  | 141,536 (40.6) | 183,072 (40.8) | 182,763 (40.7) | 182,776 (40.7) | 182,540 (40.7) | 182,690 (40.7) |
| High |  | 142,993 (41.0) | 182,907 (40.7) | 183,071 (40.8) | 183,212 (40.8) | 183,248 (40.8) | 183,396 (40.9) |
| **Fruit intake (pieces/day), N (%)** | 786 |  |  |  |  |  |  |
| <2 |  | 124,208 (27.7) | 124,481 (27.7) | 124,484 (27.7) | 124,503 (27.7) | 124,512 (27.7) | 124,490 (27.7) |
| ≥2-<5 |  | 236,989 (52.9) | 237,377 (52.9) | 237,371 (52.9) | 237,375 (52.9) | 237,358 (52.9) | 237,387 (52.9) |
| ≥5 |  | 86,953 (19.4) | 87,078 (19.4) | 87,081 (19.4) | 87,058 (19.4) | 87,066 (19.4) | 87,059 (19.4) |
| **Vegetable intake (tablespoons/day), N (%)** | 2,653 |  |  |  |  |  |  |
| <3 |  | 80,409 (17.9) | 81,021 (18.0) | 81,008 (18.0) | 81,001 (18.0) | 81,023 (18.0) | 80,997 (18.0) |
| ≥3-<6 |  | 226,427 (50.4) | 227,781 (50.7) | 227,828 (50.7) | 227,798 (50.7) | 227,798 (50.7) | 227,813 (50.7) |
| ≥6 |  | 139,447 (31.1) | 140,134 (31.2) | 140,100 (31.2) | 140,137 (31.2) | 140,115 (31.2) | 140,126 (31.2) |
| **Red meat intake, N (%)** | 3,946 |  |  |  |  |  |  |
| Never |  | 30,042 (6.8) | 30,332 (6.8) | 30,322 (6.8) | 30,312 (6.8) | 30,315 (6.8) | 30,316 (6.8) |
| Less than once a week |  | 151,250 (34.0) | 152,644 (34.0) | 152,642 (34.0) | 152,689 (34.0) | 152,665 (34.0) | 152,680 (34.0) |
| Once a week |  | 96,580 (21.7) | 97,439 (21.7) | 97,406 (21.7) | 97,379 (21.7) | 97,386 (21.7) | 97,380 (21.7) |
| ≥2 times a week |  | 167,118 (37.5) | 168,521 (37.5) | 168,566 (37.5) | 168,556 (37.5) | 168,570 (37.5) | 168,560 (37.5) |
| **Processed meat intake, N (%)** | 846 |  |  |  |  |  |  |
| Never |  | 41,367 (9.2) | 41,492 (9.2) | 41,481 (9.2) | 41,471 (9.2) | 41,479 (9.2) | 41,466 (9.2) |
| Less than once a week |  | 135,757 (30.3) | 136,019 (30.3) | 136,045 (30.3) | 136,037 (30.3) | 136,016 (30.3) | 136,023 (30.3) |
| Once a week |  | 130,930 (29.2) | 131,143 (29.2) | 131,149 (29.2) | 131,154 (29.2) | 131,175 (29.2) | 131,154 (29.2) |
| ≥2 times a week |  | 140,036 (31.3) | 140,282 (31.2) | 140,261 (31.2) | 140,274 (31.2) | 140,266 (31.2) | 140,293 (31.3) |
| **History of CRC screening, N (%)** | 7,331 |  |  |  |  |  |  |
| No |  | 306,980 (69.5) | 312,157 (69.5) | 312,198 (69.5) | 312,169 (69.5) | 312,121 (69.5) | 312,123 (69.5) |
| Yes |  | 134,625 (30.5) | 136,779 (30.5) | 136,738 (30.5) | 136,767 (30.5) | 136,815 (30.5) | 136,813 (30.5) |
| **Regular use of NSAIDs/aspirin, N (%)** | 13 |  |  |  |  |  |  |
| No |  | 311,225 (69.3) | 311,237 (69.3) | 311,234 (69.3) | 311,234 (69.3) | 311,235 (69.3) | 311,236 (69.3) |
| Yes |  | 137,698 (30.7) | 137,699 (30.7) | 137,702 (30.7) | 137,702 (30.7) | 137,701 (30.7) | 137,700 (30.7) |
| **History of mammography (women),**  **N (%)** | 384 |  |  |  |  |  |  |
| No |  | 51,063 (21.3) | 51,277 (21.4) | 51,272 (21.4) | 51,274 (21.4) | 51,282 (21.4) | 51,274 (21.4) |
| Yes |  | 188,576 (78.7) | 188,746 (78.6) | 188,751 (78.6) | 188,749 (78.6) | 188,741 (78.6) | 188,749 (78.6) |
| **History of HRT use (women), N (%)** | 813 |  |  |  |  |  |  |
| No |  | 149,064 (62.3) | 149,633 (62.3) | 149,632 (62.3) | 149,604 (62.3) | 149,633 (62.3) | 149,624 (62.3) |
| Yes |  | 90,146 (37.7) | 90,390 (37.7) | 90,391 (37.7) | 90,419 (37.7) | 90,390 (37.7) | 90,399 (37.7) |

Abbreviations: BMI = body mass index, CRC = colorectal cancer, HRT = hormonal replacement therapy, IPAQ = international physical activity questionnaire, NSAIDs = nonsteroidal anti-inflammatory drugs, SD = standard deviation.

**
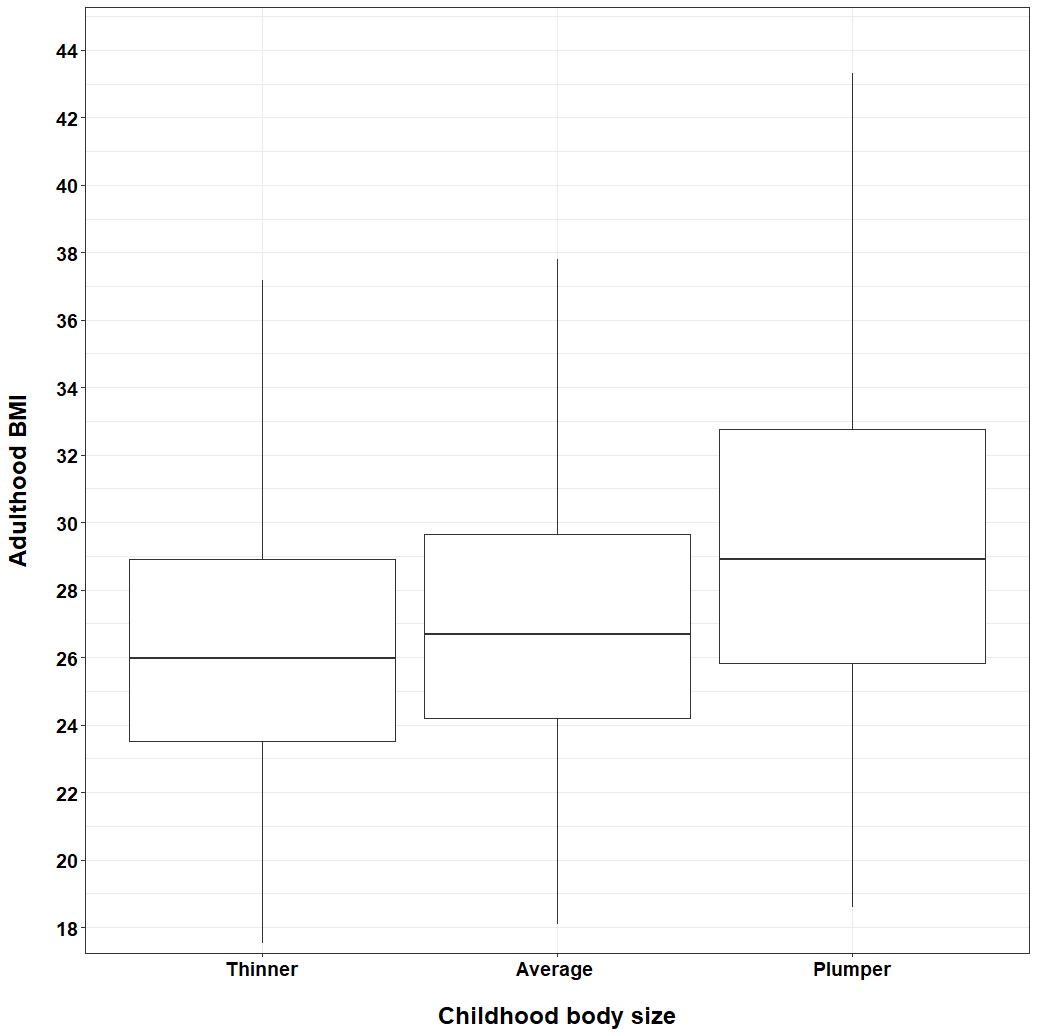
**

**Figure S1.** Adulthood BMI according to childhood body size.

Note: Lower and upper hinges correspond to 25th and 75th quantile of BMI, respectively; lower and upper whiskers correspond to 2.5th and 97.5th quantile, respectively. Outliers not shown.

Abbreviations: BMI = body mass index.

**Table S3.** Subgroup analysis based on smoking – Association of childhood body size, adulthood BMI, and body size change with obesity-related cancer risk

|  | **Never smokers*** | | **Ever smokers*** | |  |
| --- | --- | --- | --- | --- | --- |
|  | **N cases** | **HR^1^ (95% CI)** | **N cases** | **HR^1^ (95% CI)** | **p-interaction^2^** |
| **Childhood body size** | | | | | |
| Thinner | 2,623 | 1.06 (1.01-1.12) | 2,491 | 1.02 (0.97-1.07) | 0.28 |
| Average | 3,899 | Reference | 3,684 | Reference |  |
| Plumper | 1,246 | 1.08 (1.01-1.15) | 1,218 | 1.02 (0.96-1.09) |  |
| **Adulthood BMI** | | | | | |
| Normal weight | 2,457 | Reference | 1,839 | Reference | 0.090 |
| Overweight | 3,110 | 1.15 (1.09-1.21) | 3,170 | 1.21 (1.14-1.28) |  |
| Obesity | 2,201 | 1.48 (1.39-1.57) | 2,384 | 1.51 (1.42-1.61) |  |
| **Body size change** | | | | | |
| Thinner -> Normal weight | 1,022 | 1.14 (1.05-1.24) | 721 | 0.98 (0.89-1.08) | 0.022 |
| Thinner -> Overweight | 1,047 | 1.32 (1.21-1.43) | 1,089 | 1.22 (1.12-1.34) |  |
| Thinner -> Obesity | 554 | 1.59 (1.43-1.76) | 681 | 1.58 (1.43-1.75) |  |
|  |  |  |  |  |  |
| Average -> Normal weight | 1,203 | Reference | 957 | Reference |  |
| Average -> Overweight | 1,626 | 1.18 (1.10-1.28) | 1,644 | 1.18 (1.09-1.28) |  |
| Average -> Obesity | 1,070 | 1.60 (1.47-1.74) | 1,083 | 1.43 (1.31-1.56) |  |
|  |  |  |  |  |  |
| Plumper -> Normal weight | 232 | 1.11 (0.97-1.28) | 161 | 0.85 (0.72-1.01) |  |
| Plumper -> Overweight | 437 | 1.17 (1.05-1.30) | 437 | 1.11 (0.99-1.24) |  |
| Plumper -> Obesity | 577 | 1.53 (1.38-1.69) | 620 | 1.47 (1.32-1.63) |  |

*Missing data on smoking for 1,520 participants.

1 – Model adjusted for age, sex, ethnicity, height, Townsend deprivation index, education, smoking, alcohol consumption, red and processed meat consumption, 1st-degree family history of CRC and breast cancer, previous CRC screening (for obesity-related cancers and CRC), previous mammography (women only; for obesity-related cancers and breast cancer), HRT (women only), fruit and vegetable intake, NSAIDs use, and physical activity.

2 – Multivariate Wald test comparing the model with and without product terms.

Abbreviations: BMI = body mass index, CI = confidence interval, CRC = colorectal cancer, HR = hazard ratio, HRT = hormone replacement therapy, NSAIDs = nonsteroidal anti-inflammatory drugs.

**Table S4.** Age-subgroup analysis - Association of childhood body size, adulthood BMI, and body size change with obesity-related cancer risk

|  | **Age at baseline <50** | | **Age at baseline 50-59** | | **Age at baseline ≥60** | | **p-interaction^2^** |
| --- | --- | --- | --- | --- | --- | --- | --- |
|  | **N cases** | **Multivariable-adjusted HR^1^ (95% CI)** | **N cases** | **Multivariable-adjusted HR^1^ (95% CI)** | **N cases** | **Multivariable-adjusted HR^1^ (95% CI)** |  |
| **Childhood body size** | | | | | | | 0.75 |
| Thinner | 471 | 0.96 (0.86-1.08) | 1,674 | 1.08 (1.01-1.15) | 2,982 | 1.04 (0.99-1.09) |  |
| Average | 719 | Reference | 2,390 | Reference | 4,486 | Reference |  |
| Plumper | 268 | 1.01 (0.88-1.16) | 905 | 1.06 (0.98-1.14) | 1,298 | 1.03 (0.97-1.10) |  |
| **Adulthood BMI** | | | | | | |  |
| Normal weight | 543 | Reference | 1,482 | Reference | 2,274 | Reference | 0.68 |
| Overweight | 514 | 1.06 (0.94-1.20) | 1,959 | 1.21 (1.13-1.30) | 3,822 | 1.19 (1.13-1.25) |  |
| Obesity | 401 | 1.38 (1.21-1.59) | 1,528 | 1.51 (1.40-1.62) | 2,670 | 1.52 (1.43-1.61) |  |
| **Body size change** | | | | | | |  |
| Thinner -> Normal weight | 227 | 1.02 (0.85-1.21) | 601 | 1.07 (0.96-1.19) | 918 | 1.08 (0.99-1.18) | 0.93 |
| Thinner -> Overweight | 162 | 1.08 (0.88-1.31) | 676 | 1.36 (1.22-1.51) | 1,303 | 1.29 (1.19-1.40) |  |
| Thinner -> Obesity | 82 | 1.24 (0.96-1.60) | 397 | 1.65 (1.46-1.88) | 761 | 1.65 (1.50-1.81) |  |
|  |  |  |  |  |  |  |  |
| Average -> Normal weight | 270 | Reference | 735 | Reference | 1,155 | Reference |  |
| Average -> Overweight | 264 | 1.05 (0.88-1.24) | 980 | 1.19 (1.08-1.31) | 2,032 | 1.22 (1.13-1.31) |  |
| Average -> Obesity | 185 | 1.45 (1.19-1.75) | 675 | 1.49 (1.33-1.66) | 1,299 | 1.57 (1.44-1.70) |  |
|  |  |  |  |  |  |  |  |
| Plumper -> Normal weight | 46 | 0.83 (0.61-1.14) | 146 | 0.94 (0.79-1.13) | 201 | 1.05 (0.90-1.22) |  |
| Plumper -> Overweight | 88 | 1.03 (0.81-1.31) | 303 | 1.15 (1.01-1.31) | 487 | 1.17 (1.05-1.30) |  |
| Plumper -> Obesity | 134 | 1.37 (1.11-1.69) | 456 | 1.53 (1.36-1.73) | 610 | 1.50 (1.36-1.66) |  |

1 – Model adjusted for age, sex, ethnicity, height, Townsend deprivation index, education, smoking, alcohol consumption, red and processed meat consumption, 1st-degree family history of CRC and breast cancer, previous CRC screening (for obesity-related cancers and CRC), previous mammography (women only; for obesity-related cancers and breast cancer), HRT (women only), fruit and vegetable intake, NSAIDs use, and physical activity.

2 – Multivariate Wald test comparing the model with and without product terms.

Abbreviations: BMI = body mass index, CI = confidence interval, CRC = colorectal cancer, HR = hazard ratio, HRT = hormone replacement therapy, NSAIDs = nonsteroidal anti-inflammatory drugs.
